# Study of the Association Between Diets Containing Nuts and Seeds and the Degree of Abdominal Aortic Calcification

**DOI:** 10.3390/nu16244325

**Published:** 2024-12-15

**Authors:** Larry A. Tucker

**Affiliations:** College of Life Sciences, Brigham Young University, Provo, UT 84602, USA; tucker@byu.edu; Tel.: +1-801-422-4927

**Keywords:** dietary guidelines, diet, artery, endothelial, inflammation, NHANES

## Abstract

**Background**: The association between nuts and seeds (nuts/seeds) consumption and abdominal aortic calcification (AAC) has been studied rarely, if at all. However, AAC is a good marker of CVD risk and premature mortality. Consequently, the present observational study was conducted. It had two primary purposes: (1) to determine the relationship between the consumption of nuts/seeds and AAC, and (2) to examine the effects of nine potential confounding variables on the relationship between nuts/seeds consumption and AAC. **Methods**: The sample included 2611 randomly selected adults representative of the United States population. Data were collected as part of the National Health and Nutrition Examination Survey (NHANES). The outcome measure was AAC, divided into three categories: none, mild, and moderate/severe. Total consumption of nuts/seeds was assessed utilizing the mean of two diet recall assessments. **Results**: There was an inverse dose–response relationship between categories of nuts/seeds consumption and AAC, with age, sex, and race controlled (F = 6.4, *p* = 0.0233). After controlling for the demographic variables and physical activity, hypertension status, waist circumference, diabetes status, and smoking, the relationship between nuts/seeds and AAC was minimally affected (F = 6.0, *p* = 0.0268). **Conclusions**: In conclusion, nuts/seeds consumption appears to differentiate among adults with different levels of AAC. Control of many covariates had little impact on the associations. The recommendations of the U.S. Dietary Guidelines (2020–2025) that encourage the eating of nuts/seeds are supported by the results of this investigation.

## 1. Introduction

Abdominal aortic calcification, or AAC, is defined as calcium deposits that form along the abdominal section of the aorta. When AAC is present, the risk of mortality increases significantly [1,2]. Some research shows that the AAC score outperforms the Framingham Risk Score in forecasting cardiovascular outcomes in men and women, even though the Framingham Risk Score includes six classic predictors of CVD [3].

AAC is a significant risk marker of atherosclerosis, a primary component of cardiovascular disease. AAC tends to increase with age [4] and it is associated with hypertension, diabetes, smoking, obesity, and dyslipidemia [5,6,7].

Because many organs are served by the abdominal aorta, it is vital that this large artery remains healthy throughout life. Reduced blood flow within the abdominal aorta increases the risk of serious disease. According to a meta-analysis by Goncalves et al. [2], 10 studies were employed to determine the association between AAC and long-term cardiovascular outcomes. Numerous cardiovascular events served as endpoints. Based on thousands of individuals across the 10 investigations, statistical significance was reached for each of the outcomes [2]. Clearly, AAC is closely tied to cardiovascular disease and death. Chronic kidney disease, osteoporosis, and old age are significant risk factors for AAC [4]. However, in a 2024 study, He et al. concluded that some lifestyle risk factors are not significant predictors of AAC after adjusting for potential confounders [6].

Numerous studies show that a variety of dietary variables are good predictors of cardiovascular disease. This has led scientists to explore the link between diet and AAC. For example, recent research by Li and Huang et al. indicated that a plant-based diet pattern, along with protein, fiber, and other nutrients, were inversely related to AAC [8]. On the other hand, they found that caffeine consumption was unfavorably associated with AAC [8]. Hu et al. observed that lycopene was inversely tied to AAC [9], and Xiao et al. noted that saturated and polyunsaturated fats were directly tied to AAC [10].

The U.S. Dietary Guidelines (2020–2025) recommend that nutrient-dense foods be a primary focus of American diets [11]. The Guidelines specifically list nuts and seeds as healthy, nutrient-dense food choices. Moreover, the U.S. Guidelines include nuts and seeds as nutrient-dense protein foods and also as nutrient-dense oils [11]. Clearly, nuts/seeds are valuable components of a healthy dietary pattern.

Several factors make nuts/seeds cardioprotective. They contain unsaturated fatty acids, and a majority include monounsaturated fats [12]. Nuts/seeds also supply significant health-promoting bioactive compounds, including vegetable proteins and fiber, and phytochemicals, including phytosterols and phenolic metabolites [12].

Many nuts contain significant amounts of omega-3 and omega-6 fatty acids. Both of these fats have health benefits [13,14]. Research by Xiao et al. indicates that the consumption of omega-3 and omega-6 fats are favorably and inversely connected with AAC [10]. Additional research indicates that the ratio of omega-6 and omega-3 fatty acids also serves as a meaningful indicator of all-cause, cancer, and cardiovascular mortality [15]. Nuts vary in their omega-6 to omega-3 ratios, with almonds having a high ratio and walnuts having a low ratio [16].

Given these and other cardioprotective nutrients of nuts/seeds, one would expect them to protect against abdominal aortic calcification. However, to date, this gap in the literature has not been addressed. The relationship between the consumption of nuts/seeds and abdominal aortic calcification (AAC) has not been studied. Therefore, the present investigation was conducted. Its primary purpose was to determine the relationship between nuts/seeds intake and abdominal aortic calcification in a sample of 2611 men and women randomly selected from the United States population. An ancillary aim was to ascertain the extent to which potential confounding variables, such as age, sex, race/ethnicity (race), moderate physical activity, vigorous physical activity, diabetes status, waist circumference, smoking, and hypertension, impact the link between nuts/seeds and AAC.

## 2. Methods

### 2.1. Study Design and Sample

A significant strength of the National Health and Nutrition Examination Survey (NHANES) was that participants were randomly selected from the population of the United States. Therefore, outcomes can be extended to the non-institutionalized civilian population of the United States. The design of the study was cross-sectional.

For the present study, data collected by NHANES occurred over 2 years, 2013 and 2014. These were the specific years NHANES gathered information on abdominal aortic calcification (AAC). Census data were employed by NHANES to give participants specific sample weights that were used in each statistical analysis.

The United States National Center for Health Statistics authorized the data collection process and posting of the data on the Internet. No identifiable information was included in the online files, and there is no charge for the data. The official code signifying support for the data collected from 2013 to 2014 was Protocol #2011–17 [17]. All subjects gave consent to participate in the survey.

Several factors affected the number of participants in this study. NHANES collected data on individuals as young as a few months old up to 80 years and beyond. NHANES truncated age at 80 years to maximize confidentiality. There were 7574 individuals in the initial 2013–2014 sample, including individuals of all ages. The abdominal aortic calcification variable was measured only in adults 40 years old and older, decreasing the sample to 2640. Finally, men and women with missing data were not included in the sample, leaving a final sample of 2611.

### 2.2. Measurement Methods

The relationship between total nuts/seeds intake and abdominal aortic calcification (AAC) was the primary focus of this study. Age, sex, race/ethnicity, diabetes status, moderate physical activity, vigorous physical activity, waist circumference, smoking, and hypertension status were treated as covariates.

*Diet.* Nuts/seeds consumption and energy intake were each measured utilizing the mean of two 24 h diet recall assessments [18]. The details of these assessments are on the NHANES website [18]. In short, on two different occasions, one in-person and the second over the telephone, everything participants ate and drank during the previous day, from 12:00 AM to 12:00 AM, was recorded via a detailed interview with an NHANES nutrition specialist. Kilocalories for each food were calculated by NHANES utilizing information from the United States Department of Agriculture database [16,18]. Utilizing each participant’s food records, particular attention was given to the amount of nuts/seeds eaten, including peanuts. The nuts/seeds variable was reported as grams per 1000 kilocalories (kcal).

The individuals performing the dietary assessments spoke at least two languages. Scripts were used by the interviewers, and a computer afforded a standardized dietary evaluation protocol.

Findings resulting from the diet data have been evaluated, and concurrent validity has been established by numerous investigations [19,20,21]. A number of investigations have shown that NHANES diet data are closely linked to many diseases [22,23,24,25].

*Abdominal Aortic Calcification (AAC)*. NHANES AAC data first became available in September 2018 [26]. DXA examinations were conducted by skilled and licensed radiology technologists to assess the level of calcification along the abdominal aorta. According to NHANES [26], AAC can be identified on lateral spine views used to assess spine fracture risk. Sensitivity and specificity for AAC are good [27,28]. With DXA, the details of lateral spine images are comparable to the resolution of normal radiography, but DXA scans expose individuals to substantially less radiation [27,28]. Pregnant women and individuals over 450 pounds were not scanned.

The lateral spine images were obtained via Hologic Discovery model A densitometers (Hologic, Inc., Marlborough, MA, USA) and the Optasia SpinAnalyzer software [26]. The AAC-8 semi-quantitative methods for scoring were employed to report the AAC scores [29,30]. Vertebrae from L1 to L4 were used for the AAC scoring. For the present study, the AAC-8 scale included calcification of the front and back aortic walls in front of the L1-L4 vertebrae [26]. The score was reported as zero if no calcification was visible. Participants were given a score of “1” if there was calcification, and the cumulative length of the calcification was equivalent to the height of 1 vertebra or less. When the aggregate length of the calcification was equal to or more than the height of two vertebrae, subjects were given a score of “2”. Those with calcification scores of 0 were labeled “none”. Men and women with scores of 1 were categorized as “mild”, and participants with scores of 2 were categorized as “moderate/severe”.

Professional personnel evaluated the performance of the radiology technologists through in-person visits. Additional training sessions were performed with the technologists at least annually and when needed [26].

*Waist Circumference.* Trained individuals, who were evaluated regularly, performed the waist circumference measurements and were assisted by a recorder [31]. The reference position for the horizontal placement of the tape was the iliac crest. Mirrors were attached to the walls so that the correct positioning of the tape could be confirmed [31].

*Smoking.* Participants responded to multiple questions to ascertain if they had ever smoked, if they currently smoke, if they had quit smoking and similar questions. The information was used to place individuals into one of three categories: current smoker, past smoker (quit a year or more ago), and never smoked.

*Diabetes and Hypertension Status*: Subjects responded to questions asking if a doctor had ever told them that they had diabetes. They were also asked if they took medication to lower their blood sugar levels. Additionally, subjects were asked if they were taking insulin. If they answered in the affirmative to any of these three questions, they were considered diabetic. Furthermore, participants reported if they had ever been told by a physician or healthcare professional that they had hypertension or high blood pressure. Individuals reporting, yes, were classified as having hypertension or high blood pressure.

*Physical Activity.* Participants completed a questionnaire to measure physical activity [32]. Separate questions addressed moderate and vigorous activities. Recreational activities that resulted in small heart rate and breathing increases were labeled moderate. Examples of moderate physical activity were casual biking and walking. When large increases occurred in heart or breathing rates, the movement was labeled vigorous. Examples of vigorous activity included jogging, swimming, hard cycling, or walking uphill.

To quantify the time engaged in recreational physical activity, specific questions denoting moderate and vigorous activity were asked separately. Responses about days per week and duration per day were multiplied, and total time engaged in moderate and vigorous recreational activity were reported separately [32].

### 2.3. Data Analysis

The findings of the present investigation can be extended to the United States adult population because subjects were randomly selected. Consistent with this strategy, each statistical model employed person-level sample weights.

Because the sample size was large (*N* = 2611), most scientists would assume that statistical power was high. However, the degrees of freedom (*df*) were calculated by taking the number of clusters (30) minus the number of strata (15). Hence, the *df* were only 15 instead of more than 2600. In short, statistical power was modest.

The primary aim was to ascertain the link between total nuts/seeds consumption and abdominal aortic calcification. Three demographic covariates and 6 lifestyle covariates were controlled statistically to minimize their influence on the connection between total nuts/seeds intake and AAC.

The associations between AAC reported as a categorical measure (None, Mild, and Moderate/Severe) and mean nuts/seeds intake were assessed by utilizing Analysis of Variance (ANOVA). Because the nuts/seeds variable was skewed significantly, it was log-transformed, reducing the skewness index substantially from 6.5 to 1.2. Because the log of 0 cannot be computed, subjects with no intake of nuts/seeds (0 g) were assigned 1 g. The 1 g was converted to 0 g when the log-transformation was performed.

The SAS SurveyReg procedure was employed to test the differences between the nuts/seeds means across the AAC categories, with sampling weights included. Partial correlation was utilized to adjust for the covariates. SAS version 9.4 (SAS Institute, Inc., Cary, NC, USA) was the statistical package used.

## 3. Results

There were 2611 randomly selected women and men from different parts of the United States included in the sample. Their median age was 56 years, with subjects ranging from 40 to 80 years. The upper quartile of the sample reported approximately 37 min per day of activity categorized as moderate, such as walking and casual bike riding. The mean (±SE) intake of nuts/seeds per 1000 kcal was 37.0 ± 3.8 g. On average, subjects consumed 5.5 ± 0.6% of their dietary energy from nuts/seeds. Information about the continuous variables is displayed in Table 1.

The sample was mostly non-Hispanic White individuals (70.1%) followed by non-Hispanic Blacks (10.4%). Gender was nearly equally split, with 52% females and 48% males. About 12.5% of the sample reported that they had diabetes, and a total of 16% were current smokers. Almost 500 participants were determined to have moderate to severe abdominal aortic calcification (AAC), approximately 18% of the sample. Table 2 shows the traits of the sample according to the categorical measures.

Total intake of nuts/seeds per 1000 kcal differed significantly across the participants based on their degree of AAC, as shown in Table 3. The relationship was inverse and dose–response. The lower the intake of nuts/seeds, the greater the severity AAC tended to be. The association was significant after controlling for the demographic covariates and after controlling statistically for all the covariates, which included moderate and vigorous physical activity, waist circumference, smoking, blood pressure status, diabetes status, and the demographic covariates.

Consumption of nuts/seeds did not differ significantly between those with no AAC intake and mild AAC. However, the mean difference in nuts/seeds intake was substantial between those with no detectable signs of AAC and those with moderate to severe calcification, the two extreme categories. Specifically, those with no detectable aorta calcification consumed about 34% more nuts/seeds than those with moderate/severe calcification after adjusting for all the covariates (1.18 − 0.88 = 0.3, 0.3/0.88 = 0.34). The large mean difference was detected with 15 degrees of freedom.

## 4. Discussion

This investigation had three key findings: (1) Adults with moderate to severe AAC consumed significantly fewer nuts/seeds per 1000 kcal compared to those with no AAC. (2) The relation between nuts/seeds intake and severity of AAC was inverse and dose–response—the lower the intake of nuts/seeds, the more severe AAC tended to be in participants. (3) After statistically controlling all nine of the potential confounders, the strength of the relationship between nuts/seeds and AAC changed minimally.

Although the connection between eating nuts/seeds and AAC has been studied rarely if at all, several investigations have examined the impact of nuts/seeds on all-cause mortality. All-cause mortality is an excellent single measure of overall health and disease risk. Specifically, Hshieh et al. studied over 20,000 male physicians using a prospective cohort design [33]. After almost 10 years of follow-up, those with a nut intake ≥ 5 servings per week had a 26% lower death rate than those reporting no intake of nuts after controlling for numerous covariates [33]. Bao et al. also examined the risk of death across groups of women and men based on nut intake [34]. With approximately 120,000 participants, results indicated that those who ate nuts had a 7% lower mortality rate than those who consumed nuts < 1 time per week. Adults who ate nuts seven or more times per week had 20% less risk of mortality than non-consumers. Nut intake was also predictive of fewer deaths from the primary killer diseases [34].

According to the Iowa Women’s Health Study, Ellsworth et al. reported that in more than 34,000 postmenopausal women with no known cardiovascular disease, there was a 12% decreased risk of overall mortality in women who ate ≥ 2 nut servings/week compared to those who ate < 1 serving per month [35]. Finally, Grosso et al. published a meta-analysis of seven investigations indicating that one nut serving/day was related to a 27% decreased risk of all-cause mortality, and across six investigations, a 39% decreased risk of CVD mortality [1].

Why would eating nuts/seeds be inversely associated with overall mortality? And more relevant to the present investigation, why would eating nuts/seeds be inversely related to reduced abdominal aortic calcification? There are several potential mechanisms. First, nuts/seeds, including peanuts, may lower the risk of AAC by decreasing inflammation [12]. Inflammation is a critical part of the development of atherogenesis, as well as other chronic diseases. In an investigation by Jiang et al., results from the Multi-Ethnic Study of Atherosclerosis showed that the consumption of nuts/seeds was inversely related to multiple indexes of inflammation, including fibrinogen, interleukin 6, and C-reactive protein [12,36]. According to Kris-Etherton, this is significant because inflammation contributes to all stages in the progression of atherosclerosis, including the preliminary enlistment of circulating leukocytes, the triggering of endothelial dysfunction, and the rupture of atherosclerotic plaque [12].

Another mechanism could be that regular consumption of nuts/seeds might reduce AAC by decreasing unhealthy cholesterol levels. Approximately 30 years ago, Sabate, Fraser, et al. were among the first to examine the influence of diet on serum lipid levels [12]. They employed a randomized cross-over design and found that LDL levels decreased by 18% when 20% of energy intake was from walnuts [37]. Following this investigation, dozens of clinical trials were performed to evaluate the effects of nuts/seeds on cholesterol levels. These investigations have evaluated lipids, lipoproteins, and apolipoproteins [12]. Most of these studies have shown that nuts/seeds not only decrease cholesterol levels but also tend to lower cholesterol levels more than expected, given the composition of the diet [37,38,39,40].

Another mechanism that could account for the inverse relationship between nuts/seeds and AAC is the reduction of oxidative stress. Oxidative stress is the imbalance between reactive oxygen species (ROS), which result from cellular metabolism, and the body’s capacity to counteract them with antioxidants. ROS damage the endothelium or inner lining of arteries.

Nuts/seeds are good sources of tocopherols and phenolic antioxidants. According to Blomhoff et al., many nuts are among the dietary plants with the most antioxidants [41]. Moreover, because much of the fat in nuts/seeds is monounsaturated, it is not susceptible to oxidation [42]. Multiple investigations have shown that nuts/seeds reduce the oxidation of LDL molecules [40,43]. In studies incorporating nuts with high polyunsaturated content, after controlling for the saturated fat content in the meal, the reduction of oxidative stress has been consistent [39,44,45]. However, some research has failed to find a protective effect [46].

Endothelial function plays a key role in arterial health and cardiac events [47]. Endothelial damage caused by cardiovascular risk factors and atherosclerotic disease often leads to arterial abnormalities and harmful vessel responses [12,47]. Of significant importance, food can have damaging or favorable effects on these critical conditions.

Several investigations indicate that diets loaded with saturated fat tend to damage endothelial function [48,49]. Fortunately, polyunsaturated fats and other nutrients found in nuts/seeds, including antioxidants, tend to counter some of these harmful effects [50]. In a study by Ros et al., endothelial function was improved significantly in participants with high cholesterol levels who were treated with regular walnut intake [44]. The control regime focused on Mediterranean foods with similar energy and saturated fat content. Cortes et al. conducted a similar study adding walnuts to a meal containing high fat and high saturated fat [51]. The control meal was similar; only olive oil was added rather than walnuts. The walnut meal significantly improved the after-meal endothelial function compared to the olive oil meal.

Blood levels of molecules that cause cells to stick together can also serve as an index of endothelial function [47]. Research shows that diets containing meaningful amounts of alpha lipoic acid from walnuts or other foods tend to decrease plasma adhesion molecules [44,52,53]. Likewise, in a study where hamsters were fed a high-fat, atherogenic diet, walnut consumption decreased endothelial-1, a powerful endothelial stimulator [54].

Another mechanism that could contribute to the link between nuts/seeds and AAC is the length of telomeres. Telomeres are the endcaps of chromosomes and are an index of biological aging. Research by Tucker indicated that the more nuts/seeds that were eaten, the longer telomeres tended to be, signifying decreased cellular aging [21].

The current investigation had multiple limitations. First, it was an observational investigation. There was no treatment or intervention. As a result, cause-and-effect conclusions cannot be inferred. Frequent consumption of nuts/seeds could lead to less AAC. However, variables other than nuts/seeds could explain the differences found in the current study. Likewise, eating nuts/seeds could be a marker of other behaviors that might affect AAC. Many potential confounding variables were controlled, and they had minimal influence on the association between nuts/seeds intake and AAC. However, other factors not measured could account for some of the associations found in this investigation. Lastly, nuts/seeds intake was measured using two dietary recall assessments. Numerous studies have found this strategy to be valid and reliable. That is why it was used in the U.S. government-sponsored NHANES investigation. However, dietary recalls are not without measurement error. However, decreased assessment errors from the two diet recall assessments would have likely strengthened, not weakened, the association between nuts/seeds intake and AAC because measurement error is not a good predictor.

This study also had several strengths. The sample was large, with more than 2600 participants. A random selection of participants was employed so that the findings could be generalized to the adult population of the United States. Five racial/ethnic categories were included, and the subjects were 40–80 years old, so subjects represented a variety of individuals and backgrounds across the country. Intake of nuts/seeds was evaluated utilizing two 24 h diet recall assessments, not just one. Finally, those responsible for collecting the data and performing the measurements were well-trained specialists. They were evaluated frequently to safeguard against measurement errors.

## 5. Conclusions

In summary, the present study had three primary findings: (1) Adults with moderate to severe AAC consumed substantially fewer nuts/seeds compared to those with no AAC. (2) The association between nuts/seeds consumption and severity of AAC was inverse and dose–response. In short, the lower the intake of nuts/seeds among participants, the more severe AAC tended to be. (3) After adjusting for differences in all the covariates, the strength of the association between nuts/seeds and AAC changed minimally.

In conclusion, there is notable evidence from experimental trials, cross-over studies, and epidemiological investigations indicating that eating nuts/seeds improves health and reduces the risk of disease. It appears that arterial function is also improved by the regular eating of nuts/seeds, and cardiovascular events tend to be reduced significantly. The link between nuts/seeds intake and abdominal aortic calcification has rarely, if ever, been evaluated to date. However, the current investigation revealed that men and women from the United States with moderate to severe levels of AAC consume significantly fewer nuts/seeds than those with no AAC, even after being controlled statistically for many potential confounding factors. The recommendations of the Dietary Guidelines (2020–2025) produced by the United States, which recommend the regular intake of nuts/seeds, are supported by the results of the current investigation.

## Figures and Tables

**Table 1:** Key percentages and distributions of the continuous variables (*n* = 2611).

Variable	Percentile				
	10th	25th	50th	75th	90th
Age (years)	42.4	46.9	55.8	65.5	74.8
Energy intake (kcal)	1131.1	1467.7	1910.5	2399.2	2942.9
Waist (cm)	83.0	90.7	99.3	108.0	117.1
Nuts and seeds (grams/1000 kcal)	0	0	0	26.6	108.6
Moderate PA (min/day)	0	0	0	37.3	58.4
Vigorous PA (min/day)	0	0	0	0	43.5

Note. Less than 10 min of physical activity was considered 0 min by NHANES.

**Table 2:** Sample information according to the categorical variables (*n* = 2611).

Categorical Variable	*N*	%	SE
**Sex**			
Women	1356	51.9	1.50
Men	1255	48.1	1.50
**Race/Ethnicity**			
Mexican American	184	7.0	1.72
Other Hispanic	202	7.7	1.04
NH White	1828	70.1	3.33
NH Black	272	10.4	1.53
Other Race/Multiracial	125	4.8	0.90
**Diabetes**			
No	2285	87.5	1.15
Yes	326	12.5	1.15
**Smoking Status**			
Never Smoked	1481	56.7	1.78
Quit Smoking	707	27.1	1.61
Current Smoker	423	16.2	0.92
**High blood pressure**			
Yes	1164	44.6	1.83
No	1447	55.4	1.83
* AAC			
None	1888	72.3	2.23
Mild	243	9.3	1.17
Moderate/Severe	480	18.4	1.61

Note: NH is non-Hispanic. SE is the standard error of the percentage. * AAC: abdominal aortic calcification.

**Table 3:** Mean differences in total nuts/seeds consumption across categories of abdominal aortic calcification after adjusting for the covariates.

	Abdominal Aortic Calcification (*N* = 2611)							
	None		Mild		Moderate/Severe			
Outcome Variable	Mean	±SE	Mean	±SE	Mean	±SE	F	*p*
Total Nuts and Seeds Intake (g/1000 kcal)	*n* = 1893		*n* = 243		*n* = 477			
Model 1	1.29 ^a^	0.10	1.20 ^a,b^	0.18	0.91 ^b^	0.13	6.4	0.0233
Model 2	1.18 ^a^	0.08	1.14 ^a,b^	0.17	0.88 ^b^	0.12	6.0	0.0268

The outcome variable, grams of nuts/seeds eaten per 1000 kcal, was log-transformed to minimize skewness. Non-significant differences were identified by means of the same Model with the same letter. Means were modified statistically based on the covariates. Model 1 included age, sex, and race/ethnicity as covariates. Model 2 included age, sex, and race/ethnicity as covariates, plus minutes of moderate and vigorous physical activity, smoking status, waist circumference, blood pressure status, and diabetes status. The antilog of the means for Model 1 were None: 3.63, Mild: 3.31, and Moderate/Severe: 2.49. The antilog of the means for Model 2 were None: 3.27, Mild: 3.12, and Moderate/Severe: 2.41.

## Data Availability

Data for this study is posted on the NHANES website. https://wwwn.cdc.gov/nchs/nhanes/Default.aspx (accessed 27 November 2024).
